# A Review of Genetic Abnormalities in Unicentric and Multicentric Castleman Disease

**DOI:** 10.3390/biology10040251

**Published:** 2021-03-24

**Authors:** Alexandra Butzmann, Jyoti Kumar, Kaushik Sridhar, Sumanth Gollapudi, Robert S. Ohgami

**Affiliations:** 1 Agilent Technologies, Santa Clara, CA 95051, USA; 2 Department of Pathology, Stanford University, Stanford, CA 94305, USA; kumarj@stanford.edu; 3 Department of Pathology, University of California, San Francisco, CA 94143, USA; kaushik.sridhar@gladstone.ucsf.edu (K.S.); Sumanth.Gollapudi@ucsf.edu (S.G.)

**Keywords:** Castleman disease, TAFRO syndrome, POEMS syndrome, unicentric Castleman disease, multicentric Castleman disease

## Abstract

**Simple Summary:**

Castleman disease is a rare hematopoietic disorder with a broad spectrum of clinical presentations. Different subtypes have been described based on how many lymph nodes are involved, histologic appearance, and associated viral infections. Recently, significant molecular and genetic abnormalities associated with Castleman disease have been described. However, we continue to lack a framework of the biological mechanisms driving this disease process. Thus, our aim was to review all published cases of Castleman disease to date that described molecular abnormalities and correlate cytogenetic, molecular, and genetic alterations with disease subtypes. Our comprehensive review identifies subtype-specific and novel pathways which may allow for more targeted treatment options and unique biologic therapies for Castleman disease.

**Abstract:**

Castleman disease (CD) is a rare lymphoproliferative disorder known to represent at least four distinct clinicopathologic subtypes. Large advancements in our clinical and histopathologic description of these diverse diseases have been made, resulting in subtyping based on number of enlarged lymph nodes (unicentric versus multicentric), according to viral infection by human herpes virus 8 (HHV-8) and human immunodeficiency virus (HIV), and with relation to clonal plasma cells (POEMS). In recent years, significant molecular and genetic abnormalities associated with CD have been described. However, we continue to lack a foundational understanding of the biological mechanisms driving this disease process. Here, we review all cases of CD with molecular abnormalities described in the literature to date, and correlate cytogenetic, molecular, and genetic abnormalities with disease subtypes and phenotypes. Our review notes complex karyotypes in subsets of cases, specific mutations in *PDGFRB* N666S in 10% of unicentric CD (UCD) and *NCOA4* L261F in 23% of idiopathic multicentric CD (iMCD) cases. Genes affecting chromatin organization and abnormalities in methylation are seen more commonly in iMCD while abnormalities within the mitogen-activated protein kinase (MAPK) and interleukin signaling pathways are more frequent in UCD. Interestingly, there is a paucity of genetic studies evaluating HHV-8 positive multicentric CD (HHV-8+ MCD) and POEMS-associated CD. Our comprehensive review of genetic and molecular abnormalities in CD identifies subtype-specific and novel pathways which may allow for more targeted treatment options and unique biologic therapies.

## 1. Introduction

Castleman disease (CD) is an orphan disease with an incidence of 5000 new cases every year. It is classified into the clinical subtypes, unicentric Castleman disease (UCD), when only one anatomic lymph node site is affected, and multicentric Castleman disease (MCD), when multiple lymph node sites are affected. Further classification can be based on histologic features: the hyaline vascular variant (HVV), the plasma cell variant (PCV), and the mixed variant (MV). These histologic subtypes can coexist together in the same biopsy, in serial concurrent or temporally longitudinal biopsies from the same patient [1].

The UCD subtype typically is associated with a more indolent clinical presentation, with the majority of patients remaining asymptomatic. Complete remission can be achieved with surgical resection of the involved lymph node, although recurrence has been reported in rare cases [2]. In contrast, MCD can present with more severe symptoms and can be fatal in some cases. Over 50% of the MCD cases are associated with human herpesvirus 8 (HHV-8) and are classified as HHV-8 positive MCD (HHV-8+ MCD) [3]. In addition, the MCD subtype can be associated with polyneuropathy, organomegaly, endocrinopathy, monoclonal gammopathy, and skin changes (POEMS) syndrome, which is associated with monoclonal plasma cells [4]. The last clinical subtype is idiopathic MCD (iMCD), which is an extremely rare entity and has been poorly studied until recently; a subset of these patients can present with thrombocytopenia, anasarca, myelofibrosis, renal dysfunction and organomegaly. This constellation of symptoms is called the TAFRO syndrome and was described in 2012 by Iwaki et al. and Kawabata et al. [5,6]. Such cases usually have a more severe clinical course [3]. There are also aggressive cases of UCD that may fall in between a diagnosis of UCD and iMCD and may be classified as oligocentric Castleman disease (OCD) [7,8].

CD can also be associated with several malignancies, including lymphoma [9,10]. However, the relationship between lymphoma and CD, specifically iMCD, is poorly understood. iMCD diagnostic criteria indicate that iMCD should be excluded if there is a malignancy diagnosed before, concurrently, and shortly after the iMCD diagnosis since the iMCD-like pathological and clinical features are likely secondary to the malignant process [11].

Although only a few studies investigate the molecular landscape of Castleman disease, there has been a recent increase in genetic research, which has described a number of associated molecular and genetic abnormalities. These publications have discussed three possible hypotheses for the pathogenesis of CD, which include viral, clonal, or autoimmune mechanisms [4,12]. In a previous study of CD and follicular dendritic cell sarcoma by Nagy et al., evidence was provided towards a clonal pathogenesis of CD [7]. Given these significant advancements, in this study, we sought to understand the different subtypes of CD from a genetic perspective and identify common and different molecular pathogenic pathways. Here, we report on an analysis of the cytogenetic and molecular abnormalities in all CD subtypes that have been published to date.

## 2. Materials and Methods

### 2.1. Literature Search

**Figure 1:**
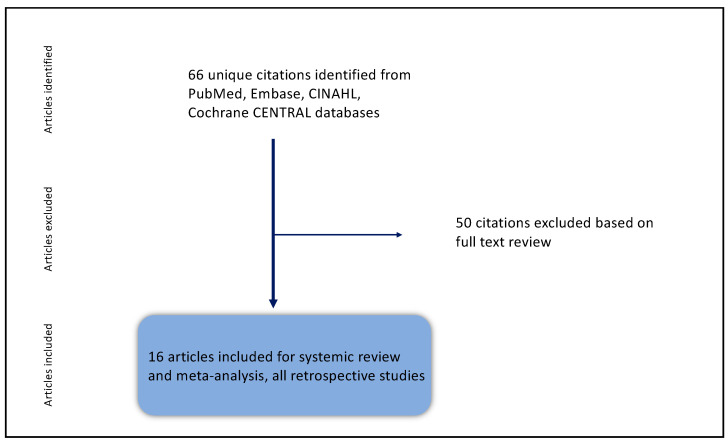
Overview of literature search workflow.

A systematic search of the literature on CD was performed and we searched common databases: PubMed, Ovid, Science Direct, and Google Scholar. Articles in other languages were included when a translated version was available. Search key terms included “multicentric Castleman disease”, “genetics Castleman disease”, “molecular Castleman disease”, “cytogenetics Castleman disease”. The search identified 66 articles, of which 16 papers were included in this analysis (Figure 1). These publications reported original data regarding the genetic aberration in CD cases. We accounted for cases reported in multiple papers and excluded any duplicates from our study as well as cases without clear subtyping or without appropriate data to allow for careful evaluation and confirmation of the diagnosis.

### 2.2. Pathway Analysis

We performed enrichment analysis for all published variants found in the included studies. The collected data were investigated with three different publicly available data browsers: EnrichR [13], GSEA [14,15], and ConsensusPathDB-human (CPDB) [16]. The pathways of CD subtypes were analyzed separately and combined. For studies that found more than 30 somatic mutations, insertion or deletions, and copy number variations, we included only the cases highlighted by the authors.

### 2.3. Data Analysis

Data were collected based on predefined criteria (sex, age, subtype, genetic changes, method of karyotypic and molecular analysis). When available, we also included clinical treatment and patient outcomes.

## 3. Results

### 3.1. Patient Cohort

The reviewed studies were published between 1993 and 2020. Overall, molecular aberrations were identified in 111 patients, which included seven cases with cytogenetic abnormalities and 104 cases with molecular data. The clinical characteristics and genetic abnormalities for each case are shown in Supplemental Tables S1 and S2 [7,17,18,19,20,21,22,23,24,25,26,27,28]. The female-to-male ratio was 21:24 for iMCD and 40:14 for UCD cases; of note, the study from Chang et al. [29] was composed entirely of female patients. The age range was 3–76 years for iMCD and 5–73 years for the UCD cases. Among all cases included in our study, 63 were UCD cases, 44 were MCD cases and four cases were POEMS-associated MCD. The treatment of the MCD cases is shown in Supplemental Tables S1 and S2. Seven of the UCD cases were treated by complete resection alone, and in five cases, patients received rituximab and/or chemo- or radiotherapy.

### 3.2. Complex Karyotypes and Abnormalities in Chromosome 7 in Castleman Disease

**Figure 2:**
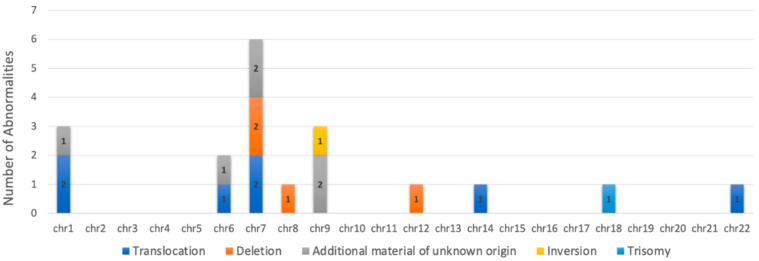
Number and type of chromosomal abnormalities seen in Castleman disease (CD) patients.

We analyzed the cytogenetic data from six patients with UCD-HVV and one patient with MCD-PC from the published literature (Supplemental Table S1). There are no studies reporting the incidence of cytogenetic changes within CD. Review of these cases demonstrated complex karyotypes for all investigated cases, including a trisomy 18 for one case of UCD-HVV (Case 7, Supplemental Table S1). An overview of the type of chromosomal abnormalities and the chromosomes affected is represented in Figure 2. Chromosomal abnormalities seen in the published CD cases included translocations, deletions, inversions, and added material of unknown origin. Of these chromosomal abnormalities, chromosome 7 was the most often appearing abnormality among the cases, including a deletion for one of the UCD-HVV cases (5/7 cases).

### 3.3. Specific Point Mutations in UCD and iMCD Discovered

**Figure 3:**
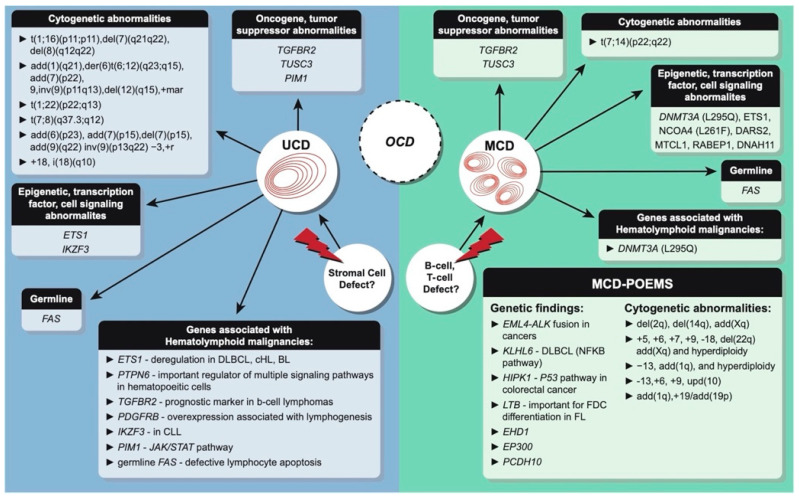
Overview of the genetic aberrations reported in 66 Castleman disease (CD) cases for both clinical CD subtypes. Key: OCD, oligocentric Castleman disease; UCD, unicentric Castleman disease; iMCD, idiopathic multicentric Castleman disease; POEMS, polyneuropathy, organomegaly, endocrinopathy, monoclonal gammopathy, and skin change; HHV-8, human herpes virus 8; DLBCL, diffuse large B-cell lymphoma; CHL, classic Hodgkin lymphoma; BL, Burkitt lymphoma; CLL, chronic lymphocytic leukemia; FDC, follicular dendritic cell; FL, follicular lymphoma.

An overview of the molecular mutational findings in CD is demonstrated in Figure 3. Among the publications identifying molecular aberrations within CD, there were two recent sequencing studies that identified specific point mutations for both UCD (*PDGFRB* N666S) and iMCD (*NCOA4* L261F). *PDGFRB* N666S was seen in 7 of 41 UCD-HVV patients (17%) and *NCOA4* L261F was identified in 4 of 22 iMCD patients (18%). Both studies reported that the mutations are specific to UCD and iMCD and have not been identified in other hematologic malignancies. The role of the specific mutations in *PDGFRB* and *NCOA4* and their impact on expression and function is discussed further in the Discussion.

### 3.4. Genes Important for Chromatin Organization Are Often Affected in iMCD

**Figure 4:**
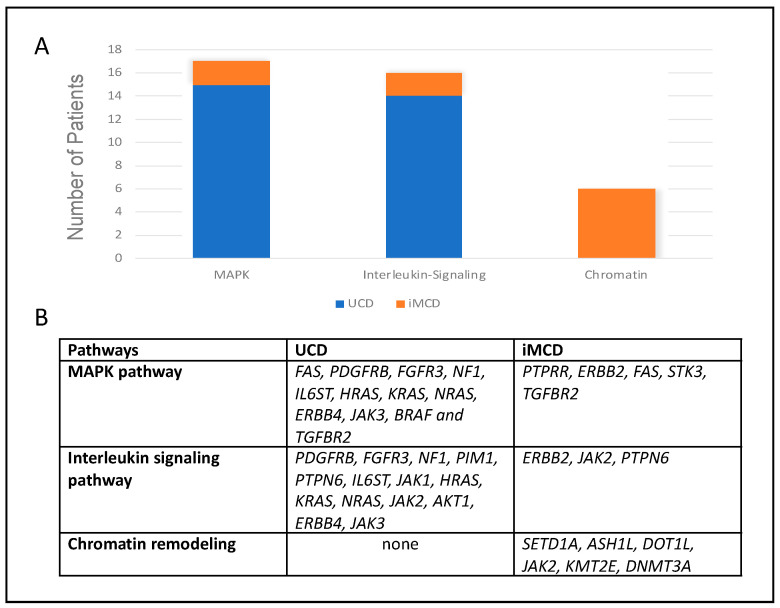
(**A**). Bar graph displaying the number of cases with gene mutations involved in the three main pathways enriched in Castleman disease (CD). (**B**). Table of the genes mutated in unicentric Castleman disease (UCD) and idiopathic multicentric Castleman disease (iMCD) that are part of the three main pathways.

We performed pathway analysis for all published molecular mutations in iMCD. Past genome-wide association study (GWAS) studies revealed that genes contributing to chromatin organization, such as *SETD1A*, *ASH1L*, *KMT2E*, and *DNMT3A*, are often affected in iMCD across all collected studies (6/35 cases) and can be seen in Figure 4. According to our analysis, mutations in these genes were the most predominant of all affected pathways (*p* = 0.03 (Reactome)).

### 3.5. MAPK Pathway and Interleukin Pathways Enriched in UCD

We performed a pathway analysis for the UCD and iMCD cases and found that the genes involved in the mitogen-activated protein kinase (MAPK) pathways (*FAS*, *PDGFRB*, *FGFR3*, *NF1*, and *TGFBR2*) were most commonly affected in UCD (*p* = 0.001 (Reactome)). Genes within the MAPK pathway were also mutated in iMCD (*PTPRR*, *ERBB2*, *FAS*, *STK3*, and *TGFBR2*) (*p* = 0.013 (Reactome)); however, the genes affecting chromatin organization were more prominent. In 14 cases of UCD, genes involved in interleukin signaling, such as *PDGFRB*, *FGFR3*, *NF1*, *PIM1*, *PTPN6*, and *IL6ST* were mutated. These findings are summarized in Figure 4.

### 3.6. HUMARA and B-Cell and T-Cell Clonality Studies in CD

Finally, we also included studies that investigated clonality in CD. B-cell and T-cell clonality were studied in both MCD and UCD cases. These studies reported 11 of 28 cases of MCD (39%) and 1 of 21 cases of UCD (4.8%) with findings of immunoglobulin heavy chain (IgH) gene arrangement (Supplemental Table S3) [8,29,30,31,32,33,34]. T-cell clonality was reported in three of four cases of MCD (75%) compared to zero of four studied UCD cases (0%). Another study by Chang et al. evaluated clonality in UCD cases by performing HUMARA [29]. They found monoclonal stromal cells in 21 of the studied 30 cases (70%).

## 4. Discussion

Our analysis examined all CD cases that reported cytogenetic, molecular, and genetic abnormalities in the context of disease subtype described in the literature to date. Through our analysis, we identified complex karyotypes and specific point mutations in *PDGFRB* in UCD and *NCOA4* in four cases of iMCD. Genetic abnormalities within interleukin signaling pathways were more frequently identified in UCD, whereas genes contributing to chromatin organization, as well as abnormalities in methylation were identified in iMCD cases. Genetic mutations in genes within the MAPK pathway were identified in both UCD and iMCD. IgH gene arrangements were noted in both UCD and MCD cases.

Chromosomal abnormalities are commonly seen in hematolymphoid neoplasms [35]. In our study, complex chromosomal aberrations were seen in all cases where a cytogenetic abnormality was seen. A study by Menke and DeWald showed chromosomal abnormalities in one of four cases. The authors state a lack of cytogenetic abnormalities in Castleman disease compared to lymphoma [36]. Of note, only seven cases with cytogenetic abnormalities were identified in our literature search, and thus, it is difficult to draw significant conclusions based on these limited number of cases. Moreover, the prevalence of cytogenetic abnormalities within CD is unknown since cytogenetic evaluation is not commonly performed in the diagnostic workup of CD. Whether all cases of CD should have cytogenetic analyses is unclear but the fact that we can see abnormalities in seven cases could argue towards consideration of increased testing in these patients.

Specifically, abnormalities involving chromosome 7 were observed in five of seven published cases that reported cytogenetic changes. Chromosome 7 is a well-studied chromosome and a number of cytogenetic aberrations involving chromosome 7 have been identified in myeloid neoplasms, T-lymphoblastic leukemia/lymphoma and splenic marginal zone lymphoma [37]. In two cases of UCD (Supplemental Table S1), chromosome 7p22 was altered, one demonstrating a translocation and another containing additional chromosomal material. *CARD11* is located at chromosome 7p22 and is known to play an important role in the NF-κB cascade [38]. Activating mutations in *CARD11* have also been described in B-cell lymphomas and in B-cell lymphocytosis and are known to drive cellular proliferation [39,40]. *CARD11* is also implicated in autoimmune lymphoproliferative disorders (ALPS) and ALPS-like syndromes, which clinically can bear some overlap with cases of CD [41]. In addition, recent published sequencing studies have identified genetic mutations associated with CD that are located on chromosome 7, such as *BRAF* (MAPK pathway, located on 7q34), *WEE2* (located on 7q34), *KMT2E* (chromatin remodeling, located on 7q22), *HDAC9* (chromatin remodeling, located on 7p21), and *DNAH11* (cell function, located on 7p15). Interestingly, *KMT2E* is involved in chromatin remodeling and may potentially be used for targeted therapy, as discussed below. Discerning the functional role of these genes in CD may help expand the understanding of this disease process.

In one case of UCD, we noted a trisomy 18 abnormality. Trisomy 18 has been previously described in non-Hodgkin lymphomas, such as extranodal marginal zone lymphoma of mucosa-associated lymphoid tissue (MALT lymphoma), follicular lymphoma, diffuse large B-cell lymphoma, and peripheral T-cell lymphoma [42,43,44,45]. Chromosome 18 includes the gene *MALT1*, which encodes a caspase-like protease that plays a role in *BCL10*-induced activation of the NF-κB pathway [46] and thus, the overexpression of *MALT1* as seen in trisomy 18 has been associated with lymphoma progression. Similarly, the only iMCD case with a cytogenetic abnormality harbored a somatic translocation 46,XY,t(7;14)(p22;q22) in lymph node tissue at the interleukin 6 (IL-6) locus (7p21–22). These findings raise the possibility that perhaps such genetic abnormalities are driven by clonal pathogenesis, analogous to malignant disease processes.

*PDGFRB* mutations have been identified across multiple malignancies. The specific point mutation *PDGFRB* N666S has not been described in hematopoietic malignancies, but has been observed in hepatocellular carcinomas (Cosmic Genomic Mutation ID: COSV55805999) [47]. One report demonstrated that *PDGFRB* N666S mutations were more frequently noted in UCD [23], while another study identified a high incidence of *PDGFRB* mutations in children with multicentric myofibroma and myofibromatosis [48]. Functional assays found the *PDGFRB* mutations to be a constitutively activating mutation, suggesting a true driver mutation effect [23,48]. Dachy et al. also reported that all cases exhibiting *PDGFRB* N666S mutations were highly sensitive to the tyrosine kinase inhibitor imatinib. Although a majority of UCD cases can be successfully treated with complete resection, surgical excision is not possible in some cases and additional treatments are needed. For cases of severe CD with mutations in *PDGFRB*, future studies should explore the application of tyrosine kinase inhibitors as they may have potential clinical utility.

*NCOA4*, also known as androgen receptor-associated protein 70 (ARA70), is a coactivator for a number of nuclear receptors. Our analysis highlighted four iMCD cases that demonstrated point mutations in *NCOA4* L261F. Previous investigations have shown that mutations in *NCOA4* play a role in carcinogenesis and increased expression of *NCOA4* was seen in various cancer cell lines [27]. You et al. described the specific point mutation L261F in *NCOA4* affected the ARA70 domain II, a highly conserved region of the gene. Interestingly, no other malignancies harboring an *NCOA4* mutation encoding L261F were identified in the literature, suggesting that perhaps *NCOA4* L261F mutations are highly specific to iMCD.

UCD and iMCD demonstrate monoclonal proliferative processes that may involve different cells of origin. Chang et al. showed that UCD is likely a monoclonal proliferation, proposing lymph node stromal cell origin [29]. Li et al. not only found recurrent *PDGFRB* N666S mutations in UCD cases, but they also investigated the cell of origin using BaseScope, a mutation-specific RNA in situ hybridization assay. Based on their results, they suggested the cell of origin in UCD is CD45 negative non-hematopoietic stromal cells, such as follicular dendritic cells (FDCs). The *PDGFRB* mutation in stromal cells likely plays an important role in UCD pathogenesis and perhaps the genome of a cell acquires a defect, such as a translocation or a driver mutation, which then leads to over proliferation of the cell with a survival advantage [23]. In contrast, there are limited studies evaluating the cell type responsible for driving iMCD pathogenesis. Potential cell types include lymphocytes, plasma cells, monocytes, endothelial cells, and FDCs; however, some studies provide evidence towards a pathogenic role of B-cells in a subset of cases [4].

Genes involved with chromatin organization were most often affected in iMCD across all studies. Specifically, *SETD1A*, *ASH1L*, and *KMT2E* are genes involved in regulating histone methyltransferase activity, while *DNMT3A* is a DNA methyltransferase. It has been previously shown that mutation or misregulation of histone methyltransferases are associated with various diseases and as a result, many histone methylation-related proteins are being studied as potential therapeutic targets [49].

Genetic abnormalities within the MAPK and interleukin signaling pathways were more frequently identified in UCD, while genes within the MAPK pathway were also mutated in iMCD. These findings suggest that perhaps UCD and iMCD both share similar pathways of MAPK pathogenesis and can be distinguished by interleukin and cytokine regulation. Studies have described IL-6 as a pathological driver in CD; however, the involved signaling pathways remain largely unknown [50]. NF-κB signaling is the primary transcription factor involved in IL-6 and the MAPK signaling pathway is primarily activated by IL-6 [50]. IL-6 is a multifunctional cytokine that promotes B-cell and plasma cell maturation, mediates inflammatory response, and induces secretion of vascular endothelial growth factor (VEGF). Monoclonal antibodies targeting IL-6 (siltuximab) and the IL-6 receptor (tocilizumab) have been developed for patients with iMCD. Interestingly, there are patients with iMCD who fail to respond to siltuximab, highlighting the importance of identifying pathways other than IL-6/IL-6 receptor signaling and further understanding the precise cell types in which these signaling pathways are dysregulated.

Interestingly, the role of HHV-8 in the pathogenesis of HHV-8+ MCD involves upregulation of NF-κB by latently expressed viral Fas-associating protein with death domain-like interleukin-1-converting enzyme (viral-FLICE) inhibitory protein or viral microRNA-K1 [51]. In addition, HHV-8 causes upregulation of VEGF and other factors using a viral G-protein couple receptor [51]. It has been found that secreted factors induce B-cell and plasma cell proliferation, angiogenesis, and an acute-phase reaction. In some cases, B immunoblasts infected with HHV-8 are highly proliferative, which permits accumulation of newly acquired mutations within the genomes of the infected cells. Ultimately, clones harboring mutations that enhance growth and survival have a competitive advantage compared to other clones, allowing transformation into lymphoma.

Studies investigating clonality using HUMARA and B- and T-cell clonality studies were also included in our analysis. Although one case of UCD [8,31] and 11 cases of MCD [8,30,32,33,52] with B-cell gene arrangements were identified, most studies investigated only a limited number of cases. There were two studies that included larger cohorts (34 and 20 cases), but most of the cases were negative for B-cell clonality [30,31]. The studies that identified immunoglobulin (Ig) rearrangements reported a low burden of B-cell gene rearrangements by Southern blot, which may explain the number of undetected rearrangements in other cases. Chang et al. performed HUMARA on 30 UCD cases and found monoclonal stromal cells in 21 of them. With B-cell clonality in MCD and stromal cell clonality shown in UCD, these studies point towards different cells of origin for the CD subtypes.

The molecular data collected in our study point toward UCD and iMCD representing distinct genetic landscapes and arising from different cells. However, the MAPK pathway appears to be affected in both CD subtypes, likely accounting for the similar phenotypes found in both UCD and iMCD (hyaline-vascular, plasma cell type, and mixed variant), which can occur simultaneously in one patient [1]. Moreover, overlapping molecular changes may also explain those UCD cases with more severe clinical manifestations [7,8]. These aggressive cases of UCD may fall in between a diagnosis of UCD and iMCD and may be classified as OCD (Figure 3). One recent study published the molecular findings of only three iMCD-TAFRO cases, which is the CD subtype with the most severe clinical presentation. Further molecular studies are necessary to better understand the different subtypes of CD.

Our analysis was limited by the paucity of genetic studies evaluating all subtypes of Castleman disease, but especially HHV-8+ MCD and POEMS-associated CD. As a result, conclusions were difficult to draw for these subtypes and additional work is required to further examine these entities. Additionally, given the limited availability of long-term outcome data in the published literature, quantitative analysis of survival was not possible. Currently it is unknown whether mutations in genes within the chromatin, MAPK, and interleukin signaling pathways impact survival or treatment response in patients with CD. Future studies are warranted to explore correlations between mutation status and clinical outcome in CD.

The data underlying our analysis were generated by different sequencing methods, such as whole genome sequencing, whole exome sequencing, and targeted sequencing, as well as more traditional methods to investigate genetic/chromosomal abnormalities. This may cause a bias in the reflection of the genetic landscape since a targeted panel, for example, may not detect the variants found in whole genome or exome sequencing, depending on the design. Further, sequencing and variant analysis methods can vary from group to group, making it challenging to compare different studies with each other. A possible solution would be the requirement to publish more data with the results, such as a detailed description of the sequencing and analysis method, more information regarding the variants found, and setting quality thresholds for the sequencing data.

## 5. Conclusions

In summary, recent evidence has improved our understanding of CD, leading to greater insight towards the genetic landscape of this disease process. Future studies should seek to expand on these investigations to further elucidate the underlying pathogenesis of this heterogeneous disease process and help guide therapeutic management.

## Supplementary Materials

The following are available online at https://www.mdpi.com/2079-7737/10/4/251/s1, Table S1: Previously reported cytogenetic data, Table S2: Previously reported molecular mutation data., Table S3: Reports of clonality in CD.
