# Supplementary material for: A Review of Genetic Abnormalities in Unicentric and Multicentric Castleman Disease
Source: Biology (Basel). 2021 Mar 24;10(4):251. doi: 10.3390/biology10040251 (PMC8063830; doi:10.3390/biology10040251)
Supplement: Supplementary file 1 [file biology-10-00251-s001.pdf]

Supplemental Table S1. Previously reported cytogenetic data.

Case	Type of CD	Age	Sex	Genetic changes	Method	Size of lesion (cm)	Treatment	Outcome/Survival
Case 1 *	MCD	69	M	46,XY,t(7;14)(p22;q22)	NA	NA	Prednisolone + melphalan	Decreasing IL-6, Improvement anemia, lymph node swelling + hypergammaglobulinemia
Case 2 +	UCD-HVV	36	F	46,XX,t(1;16)(p11;p11), del(7)(q21q22),del(8)(q12q22) [15:20]	Short term suspension culture, G-band karyotyping	5	Complete resection	NED after 12 months
Case 3 Φ	UCD-HVV	34	F	46,XX,add(1)(q21),der(6)t(6;12)(q23;q15), add(7)(p22),-9,inv(9)(p11q13),del(12)(q15),+mar	Short term suspension culture, G-band karyotyping	2-3	Complete resection	NED after 11 months
Case 4 #	UCD-HVV	12	F	46,XX,t(1;22)(p22;q13) [18:20]		5	Complete resection	NED after 10 months
Case 5 #	UCD-HVV	39	M	46,XY,t(7;8)(q37.3;q12) [1:20]		5.2	Complete resection	NA
Case 6 ∇	UCD-PCV	35	M	46,XY,add(6)(p23), add(7)(p15),del(7)(p15),add(9)(q22),inv(9)(p13q22) −3,+r	In situ culture, karyotyping	5-6	Complete resection	NED after 24 months
Case 7 ǀ	UCD-HVV	69	M	46, X,-Y, +18, i(18)(q10) [8:20]	Short term suspension culture, G-band karyotyping	2	Complete resection + Rituximab	NA

F, female; M, male; iMCD, idiopathic multicentric Castleman disease; UCD, unicentric Castleman disease; UCD-HVV, UCD-hyaline vascular variant; UCD-PCV, UCD-plasma cell variant; NED, no evidence of disease; NA, not available. * Nakamura et al. 1993 [17]. + Pauwels et al. 2000 [18]. Φ Cokelaere et al. 2002 [19]. # Chen et al. 2006 [20]. ∇ Reichard et al. 2011 [21]. ǀ Kojima et al. 2018 [22].

Supplemental Table S2. Previously reported molecular mutation data.

Case	Type of CD	Age	Sex	Genetic findings	Method	Size of lesion(cm)	Treatment	Outcome/ survival
Case 8[+]	iMCD-PC	35	M	*DNMT3A*(L295Q) Copy number gain: *ETS1, PTPN6, TGFBR2, TUSC3*	Targeted deep sequencing	2-5	Complete resection + Rituximab-Etoposide/Sil-tuximab	Alive after 2 yrs
Case 9[+]	UCD-MV	59	F	Copy number gains: *ETS1, PTPN6, TGFBR2, IKZF3, PIM1*	Targeted deep sequencing	5-13.6	Complete resection + tocilizumab	Alive after 4 yrs
Case 10[+]	UCD-HVV	36	M	Copy number gains in HIST1 cluster	Targeted deep sequencing	4.5-12	Complete resection + XRT	Alive after 1 yr
Case 11[+]	FDCS from UCD-HVV	27	M	*CABIN1, IL17RD, NUDT4* Copy number gain: *MAP3K5* Copy number loss: *FANCB, BRAF, JAK2, MSH3, NFKB1*	WES	NA	Complete resection	Alive after 3 yrs
Case 12[+]	FDCS from UCD-HVV	73	F	*TOP3B, ZBTB7A, DRD5* Copy number gain: *AR, MAP3K15, MED12, FANCB* copy number loss: *HIST1 cluster, BRAF, JAK2, NPM1*	WES	NA	NA	NA
Case 13[+]	FDCS from UCD-HVV	55	M	*NCAPH, SETD2, WDR55, STAT3, MARCH7* Copy number gain: VCAM1	WES	8-14	Complete resection + CHOP	Alive after 2 yrs
Case 14[ö]	UCD-HVV	N/A	N/A	*PDGFRB* (N666S)	Targeted deep sequencing	NA	NA	NA

Case 15[ö]	UCD-HVV	N/A	N/A	*PDGFRB* (N666S)	Targeted deep sequencing	NA	NA	NA
Case 16[ö]	UCD-HVV	N/A	N/A	*PDGFRB* (N666S)	Targeted deep sequencing	NA	NA	NA
Case 17[ö]	UCD-HVV	N/A	N/A	*PDGFRB* (N666S)	Targeted deep sequencing	NA	NA	NA
Case 18[ö]	UCD-HVV	20-50	F	*PDGFRB* (N666S), *IL6ST*, *CDC27*	WES, targeted deep sequencing	NA	NA	NA
Case 19[ö]	UCD-HVV	20-50	M	*PDGFRB* (N666S)	WES, targeted deep sequencing	NA	NA	NA
Case 20[ö]	UCD-HVV	20-50	F	*PDGFRB* (N666S)	WES, targeted deep sequencing	NA	NA	NA
Case 21[ö]	UCD-HVV	20-50	M	*ALK*	WES	NA	NA	NA
Case 22[ö]	UCD-HVV	20-50	M	*FGFR3, SET1B, FAT2, WEE2, NF1*	WES	NA	NA	NA
Case 23[ö]	UCD-HVV	20-50	F	*CHD5*	WES	NA	NA	NA
Case 24[ö]	UCD-HVV	>50	F	*BAZ1A*	WES	NA	NA	NA
Case 25[ö]	UCD-HVV	>50	M	*MSL3*	WES	NA	NA	NA
Case 26[ö]	UCD-HVV	20-50	M	*MAP3K10*	WES	NA	NA	NA
Case 27[ö]	iMCD-HV	20-50	F	*ALK*	WES	NA	NA	NA
Case 28[ö]	iMCD-PC	20-50	M	*ROS1, FAT3, RBL1*	WES	NA	NA	NA
Case 29[ö]	iMCD-HV	20-50	F	*ERBB2, FRK, BAZ1A, KMT2E, JAK2, STK3, LATS2, PTPRR*	WES	NA	NA	NA
Case 30[ö]	iMCD-PC	>50	F	*BCOR, HDAC9, CDC27*	WES	NA	NA	NA
Case 31[ö]	iMCD-PC	20-50	M	*DOT1L*	WES	NA	NA	NA
Case 32[ö]	iMCD-PC	20-50	F	*SETD1A*	WES	NA	NA	NA
Case 33[ö]	iMCD-PC	>50	F	*ASH1L*	WES	NA	NA	NA
Case 34[ö]	iMCD-PC	>50	F	*IDH1, LATS2*	WES	NA	NA	NA
Case 35[ǀ, ◦]	iMCD-M	23	M	*FAS* (R68G), germline	WGS	9-12	8 Cycles Rituximab + 4 cycles consolidation	CR for 14 month, relapse at 28 +33 yrs old
Case 36[ǀ]	UCD-HVV	62	M	*FAS* (R68G), germline	WGS	NA	Complete resection	NA

Case 37[e]	UCD-HVV	17-69	NA	*NRAS, STK11, KRAS*	Targeted Sequencing	NA	Complete resection	CR for median follow-up of 78 months
Case 38[e]	UCD-HVV	17-69	NA	*JAK2, SMAD4*	Targeted Sequencing	NA	Complete resection	CR for median follow-up of 78 months
Case 39[e]	UCD-HVV	17-69	NA	*MLH1, AKT1, ERBB4*	Targeted Sequencing	NA	Complete resection	CR for median follow-up of 78 months
Case 40[e]	UCD-HVV	17-69	NA	*VHL, HRAS, STK11*	Targeted Sequencing	NA	Complete resection	CR for median follow-up of 78 months
Case 41[e]	UCD-HVV	17-69	NA	*JAK3*	Targeted Sequencing	NA	Complete resection	CR for median follow-up of 78 months
Case 42[à]	iMCD-PC	64	F		WES	NA	Prednisone	Alive after 101 months
Case 43[à]	iMCD-HV	56	M		WES	NA	COP	Alive after 84 months
Case 44[à]	iMCD-PC	49	M		WES	NA	COP	Alive after 78 months
Case 45[à]	iMCD-HV	76	M		WES	NA	COP	Deceased after 8 months
Case 46[à]	iMCD-HV	29	M		WES	NA	COP	Deceased after 29 months
Case 47[à]	iMCD-PC	43	F		WES	NA	E-COP	Deceased after 1 month
Case 48[à]	iMCD-HV	57	M		WES	NA	CHP	Alive after 63 months
Case 49[à]	iMCD-PC	45	F		WES	NA	Supportive Care	Deceased after 1 month
Case 50[à]	iMCD-PC	44	M		WES	NA	CHOP	Deceased after 21 months
Case 51[à]	iMCD-PC	57	M		WES	NA	Prednisone	Deceased after 3 months
Case 52[à]	iMCD-PC	52	M		WES	NA	R2-CHOP	Alive after 53 months
Case 53[à]	iMCD-PC	66	F		WES	NA	Prednisone	Alive after 56 months
Case 54[à]	iMCD-PC	67	M		WES	NA	Prednisone	Deceased after 19 months
Case 55[à]	iMCD-PC	63	F	*Overall 7195 non-silent somatic mutations (NCOA4*	WES	NA	Prednisone	Alive after 43 months

				(L261F), DARS2, MTCL1, RABEP1 and DNAH11 associated with unfavorable diagnosis)				
Case 56[à]	iMCD-PC	67	F		WES	NA	Prednisone	Alive after 42 months
Case 57[à]	iMCD-PC	42	F		WES	NA	Prednisone	Alive after 40 months
Case 58[à]	iMCD-PC	65	M		WES	NA	COP+thali-domide	Deceased after 1 month
Case 59[à]	iMCD-PC	37	F		WES	NA	CHOP	Alive after 31 months
Case 60[à]	iMCD-PC	69	M		WES	NA	CHOP	Alive after 28 months
Case 61[à]	iMCD-PC	35	F		WES	NA	Prednisone	Alive after 28 months
Case 62[à]	iMCD-PC	67	F		WES	NA	Prednisone	Alive after 28 months
Case 63[à]	iMCD-PC	31	F		WES	NA	PD	Alive after 25 months
Case 64[2]	iMCD-TAFRO	32	M	*MEK2* (P128L)	Targeted Sequencing	NA	Steroide+ Tocilizumab	Recovered but relapsed
Case 65[2]	iMCD-TAFRO	3	M	*RUNX1* (G60C), germline	Targeted Sequencing	NA	Steroide+ Tocilizumab	Recovered, no relapse for 3 years
Case 66[2]	iMCD-TAFRO	20	M	uncertain findings (*RARA*, *SETD2*)	Targeted Sequencing	NA	Steroide + Siltuximab	Recovered

F, female; M, male; UCD, unicentric Castleman disease; iMCD, idiopathic multicentric Castleman disease; UCD-HVV, UCD-hyaline vascular variant; UCD-PCV, UCD-plasma cell variant; FDCS, follicular dendritic cell sarcoma; iMCD-PC, iMCD-plasma cell variant; UCD-MV, UCD-mixed variant; iMCD-HV, iMCD-hyaline vascular variant; iMCD-M, iMCD-mixed variant; iMCD-TAFRO, iMCD- thrombocytopenia, anasarca, myelofibrosis, renal dysfunction, and organomegaly; PCR, polymerase chain reaction; HIV, human immunodeficiency virus; WES, whole exome sequencing; WGS, whole genome sequencing; CHOP, cyclophosphamide, doxorubicin, vincristine, and prednisone; CHP, cyclophosphamide, doxorubicin, prednisone; COP, cyclophosphamide, doxorubicin, vincristine; E-COP, etoposide-COP; R2-CHOP, rituximab, lenalidomide + CHOP; NA, not available; yrs, years. [+] Nagy et al. 2018 [7]. [φ] Li et al. 2019 [23]. [ǀ] Baker et al. 2018 [24]. [°]Ocio et al. 2005 [25]. [ℇ]Legras et al. 2018 [26]. [◊]You et al. 2019 [27]. [″]Yoshimi et al. 2020 [28].

Supplemental Table S3. Reports of clonality in CD.

Case	Type of CD	Age	Sex	Genetic findings	Method	Size of lesion(cm)	Treatment	Outcome/ survival
Case 67[ς]	UCD-HVV	59	F	Monoclonal	HUMARA	2.5	NA	NA
Case 68[ς]	UCD-HVV	43	F	Monoclonal	HUMARA	11	NA	NA
Case 69[ς]	UCD-HVV	12	F	Polyclonal	HUMARA	4	NA	NA
Case 70[ς]	UCD-HVV	21	F	Polyclonal	HUMARA	4	NA	NA
Case 71[ς]	UCD-HVV	45	F	Monoclonal	HUMARA	5	NA	NA
Case 72[ς]	UCD-PCV	55	F	Monoclonal	HUMARA	4	NA	NA
Case 73[ς]	UCD-HVV	65	F	Monoclonal	HUMARA	5.9	NA	NA
Case 74[ς]	UCD-HVV	36	F	Polyclonal	HUMARA	2.3	NA	NA
Case 75[ς]	UCD-HVV	19	F	Monoclonal	HUMARA	5.1	NA	NA
Case 76[ς]	UCD-HVV	29	F	Polyclonal	HUMARA	7.5	NA	NA
Case 77[ς]	UCD-HVV	40	F	Polyclonal	HUMARA	NA	NA	NA
Case 78[ς]	UCD-PCV	39	F	Monoclonal	Methylation specific PCR	NA	NA	NA
Case 79[ς]	UCD-HVV	49	F	Monoclonal	HUMARA	NA	NA	NA
Case 80[ς]	UCD-HVV	12	F	Monoclonal	Methylation specific PCR	5	NA	NA
Case 81[ς]	UCD-HVV	5	F	Monoclonal	Methylation specific PCR	1.5	NA	NA
Case 82[ς]	UCD-HVV	45	F	Monoclonal	Methylation specific PCR	NA	NA	NA
Case 83[ς]	UCD-HVV	35	F	Monoclonal	Methylation specific PCR	NA	NA	NA
Case 84[ς]	UCD-HVV	61	F	Monoclonal	Methylation specific PCR	NA	NA	NA
Case 85[ς]	FDCS from UCD-HVV	25	F	Monoclonal	Methylation specific PCR	NA	NA	NA
Case 86[ς]	UCD-HVV	21	F	Polyclonal	HUMARA	7.5	NA	NA
Case 87[ς]	UCD-HVV	45	F	Monoclonal	HUMARA	6.5	NA	NA
Case 88[ς]	UCD-PCV	27	F	Monoclonal	HUMARA	4	NA	NA
Case 89[ς]	UCD-HVV	31	F	Monoclonal	HUMARA	8	NA	NA
Case 90[ς]	UCD-HVV	48	F	Polyclonal	HUMARA	2.2	NA	NA
Case 91[ς]	UCD-HVV	8	F	Polyclonal	Methylation specific PCR	5	NA	NA
Case 92[ς]	UCD-HVV	37	F	Polyclonal	HUMARA	1.6	NA	NA
Case 93[ς]	UCD-HVV	31	F	Monoclonal	HUMARA	4	NA	NA
Case 94[ς]	UCD-HVV	19	F	Monoclonal	HUMARA	4.1	NA	NA
Case 95[ς]	UCD-HVV	43	F	Monoclonal	Methylation specific PCR	8.5	NA	NA
Case 96[ς]	UCD-HVV	14	F	Monoclonal	Methylation specific PCR	14	NA	NA
Case 97[ς]	UCD-HVV	30	F	Polyclonal	HUMARA	2.5	NA	NA
Case 98[c]	MCD-M	41	M	Major Ig gene	Southern Blot	NA	NA	NA

				rearrangement				
Case 99[c]	MCD-HV	61	M	Ig gene rearrangement	Southern Blot, PCR	NA	NA	NA
Case 100[c]	MCD-HV	71	F	Minor Ig gene rearrangement	PCR	NA	NA	NA
Case 101[c]	MCD-M	64	F	Major Ig gene rearrangement	Southern Blot, PCR	NA	Chemotherapy	CR, Relapse after 8 yrs
Case 102[h]	UCD-PCV	65	M	Ig gene rearrangement	PCR	NA	NA	NA
Case 103[m]	MCD-PC*	29	F	Ig gene rearrangement/ T-cell rearrangement	Southern Blot	NA	Prednisone	Remission and recurrences
Case 104[m]	MCD-PC*	62	M	Ig gene rearrangement/ T-cell rearrangement	Southern Blot	NA	Prednisone	Remission and recurrence
Case 105[m]	MCD-PC*	67	M	Ig gene rearrangement/ T-cell rearrangement	Southern Blot	NA	Prednisone	Remission and recurrences
Case 106[u]	POEMS associated MCD*	24	M	Ig gene rearrangement	Southern Blot	NA	High dose melphalan and cyclophos-phamide	Disease stable, alive after 7 yrs
Case 107[u]	POEMS associated MCD*	52	M	Ig gene rearrangement	Southern Blot	NA	NA	Died after 3 yrs
Case 108[u]	POEMS associated MCD*	41	M	Ig gene rearrangement	Southern Blot	NA	Complete resection	Disease stable, alive after 2 yrs
Case 109[Δ]	POEMS associated MCD	28	M	Ig gene rearrangement	Southern Blot	NA	Steroids and cytotoxic therapy	Died after 3 yrs
Case 110[±]	HIV positive iMCD-PC	49	F	Incomplete Ig gene rearrangement	PCR	NA	Rituximab and Etoposide	Clinical improvement within 4 months

* no HIV, Kaposi sarcoma or lymphoma was reported in these cases. F, female; M, male; UCD, unicentric Castleman disease; iMCD, idiopathic multicentric Castleman disease; UCD-HVV, UCD-hyaline vascular variant; UCD-PCV, UCD-plasma cell variant; FDCS, follicular dendritic cell sarcoma; iMCD-PC, iMCD-plasma cell variant; UCD-MV, UCD-mixed variant; iMCD-HV, iMCD-hyaline vascular variant; iMCD-M, iMCD-mixed variant; iMCD-TAFRO, iMCD- thrombocytopenia, anasarca, myelofibrosis, renal dysfunction, and organomegaly; Ig, immunoglobulin; PCR, polymerase chain reaction; HIV, human immunodeficiency virus; WES, whole exome sequencing; WGS, whole genome sequencing; CHOP, cyclophosphamide, doxorubicin, vincristine, and prednisone; CHP, cyclophosphamide, doxorubicin, prednisone; COP, cyclophosphamide, doxorubicin, vincristine; E-COP, etoposide-COP; R2-CHOP, rituximab, lenalidomide + CHOP; NA, not available; yrs, years, POEMS, polyneuropathy, organomegaly,

endocrinopathy, monoclonal-protein, skin changes. [ς] Chang et al. 2014 [29]. [χ]Soulier et al. 1995 [30]. [η]Al-Maghrabi et al. 2006 [31]. [μ]Hanson et al. 1988 [32]. [Δ]Gould et al. 1990 [33]. [υ]Hall et al. 1989 [8]. [±]Goyal et al. 2015 [34].
